# Immune response induced by a *Streptococcus suis* multi-serotype autogenous vaccine used in sows to protect post-weaned piglets

**DOI:** 10.1186/s13567-024-01313-x

**Published:** 2024-05-07

**Authors:** Alison Jeffery, Mélina Gilbert, Lorelei Corsaut, Annie Gaudreau, Milan R. Obradovic, Simon Cloutier, Marie-Christine Frenette, Charles Surprenant, Sonia Lacouture, Jose Luis Arnal, Marcelo Gottschalk, Mariela Segura

**Affiliations:** 1 Swine and Poultry Infectious Diseases Research Centre, and Research Group On Infectious Diseases in Production Animals, Faculty of Veterinary Medicine, University of Montreal, St-Hyacinthe, QC J2S 2M2 Canada; 2 S.V.A. Triple-V Inc., Acton Vale, QC J0H 1A0 Canada; 3 F. Ménard, Ange-Gardien, QC J0E 1E0 Canada; 4 Exopol, Veterinary Diagnostic and Autogenous Vaccine Laboratory, Zaragoza, Spain; 5 Present Address: Vaccine and Infectious Disease Organization, University of Saskatchewan, Saskatoon, SK Canada

**Keywords:** *Streptococcus suis*, multiserotype autogenous vaccines, sows, bacterin, maternal antibodies, protection

## Abstract

*Streptococcus suis* is a bacterial pathogen that causes important economic losses to the swine industry worldwide. Since there are no current commercial vaccines, the use of autogenous vaccines applied to gilts/sows to enhance transfer of passive immunity is an attractive alternative to protect weaned piglets. However, there is no universal standardization in the production of autogenous vaccines and the vaccine formulation may be highly different among licenced manufacturing laboratories. In the present study, an autogenous vaccine that included *S. suis* serotypes 2, 1/2, 5, 7 and 14 was prepared by a licensed laboratory and administrated to gilts using a three-dose program prior to farrowing. The antibody response in gilts as well as the passive transfer of antibodies to piglets was then evaluated. In divergence with previously published data with an autogenous vaccine produced by a different company, the increased response seen in gilts was sufficient to improve maternal antibody transfer to piglets up to 5 weeks of age. However, piglets would still remain susceptible to *S. suis* disease which often appears during the second part of the nursery period. Vaccination did not affect the shedding of *S. suis* (as well as that of the specific *S. suis* serotypes included in the vaccine) by either gilts or piglets. Although all antibiotic treatments were absent during the trial, the clinical protective effect of the vaccination program with the autogenous vaccine could not be evaluated, since limited *S. suis* cases were present during the trial, confirming the need for a complete evaluation of the clinical protection that must include laboratory confirmation of the aetiological agent involved in the presence of *S. suis*-associated clinical signs. Further studies to evaluate the usefulness of gilt/sow vaccination with autogenous vaccines to protect nursery piglets should be done.

## Introduction

*Streptococcus suis* is a bacterial pathogen that causes important economic losses to the swine industry worldwide. It affects mostly post-weaned piglets, causing mainly arthritis, meningitis, polyserositis, endocarditis and septicemia with sudden death [1]. A total of 35 serotypes had originally been described, although six of them have more recently been re-classified within other streptococcal species [2]. In Europe, few serotypes (mostly serotypes 2 and 9) are frequently recovered from diseased animals [3]. However, in North America, although the most prevalent serotypes isolated from diseased animals are 1/2 and 2, other serotypes are also routinely isolated from diseased pigs in both Canada and the United States [4]. Isolates belonging to more than one serotype are also commonly recovered from diseased piglets within a single farm in North America [5]. This may be explained, at least in part, by the co-infection with the porcine reproductive and respiratory syndrome virus (PRRSV), which is known to render pigs more susceptible to *S. suis* disease [1]. Control of *S. suis* infections in swine productions is also important as it has been reported to be an emerging zoonotic pathogen. There is an increased risk attaining to those who have close contact with infected pigs or pork-derived products, such as pig producers and employees, butchers, meat inspectors, and swine veterinarians in Western countries [1, 3].

*S. suis* epidemiology is complex (multiple strains, multiple serotypes with a high phenotypic diversity) and difficulties in disease control and management are commonly reported in the field [6]. Different factors can contribute to development of the disease including immune status of the herd, arrival of a new virulent strain to the herd, co-current infections, quality of the environment and other management factors leading to stress [7]. Management practices, such as early medicated and segregated early weaning, do not eliminate *S. suis* infections, since piglets are infected very early in life or even during farrowing [1]. Antimicrobials have been used (and still are where allowed) for metaphylactic and/or prophylactic treatment. However, there has been increasing concern worldwide around antimicrobial use to control *S. suis* infections [8]. High rates of resistance to macrolides/lincosamides and tetracyclines are observed and attributed to the heavy use of antimicrobials in swine [8]. Indeed, *S. suis* is an important antimicrobial resistance reservoir, with a high risk of transmission to other veterinary and human pathogens, due to the presence of mobile genetic elements carrying resistance genes transferable at high frequency within the species, as well, between bacterial species [9]. Until recently, *S. suis* has been considered as being susceptible to penicillin and amoxicillin, extensively used to treat these infections. However, recent data showed increased resistance to these antibiotics [10].

*S. suis* disease prevention should shift to focus on the management of the predisposing factors and, mainly, vaccines. A commercial multivalent efficacious vaccine has not been developed so far, probably due to the high number of serotypes (with currently not proven cross-protection), and high genetic variation amongst strains within the same serotype [6]. The use of bacterial autogenous vaccines (bacterins) has increased in popularity as these vaccines are relatively of low cost for swine producers and can include several serotypes in one vaccine formulation. These vaccines are composed by the isolate(s) recovered from diseased pigs within a farm and produced by an accredited manufacturing laboratory, and then applied to the original farm [11]. Field studies evaluating the protective capacity of autogenous vaccines produced by licenced laboratories are limited and presented contradictory results [11–15]. Absence of protective responses from these vaccines have been attributed to the failure of whole-bacterial antigens to elicit an immune response due the inactivation processing, production of antibodies to antigens not associated with protection, and/or the use of inappropriate adjuvants [6, 11, 16]. Indeed, it is difficult to compare published studies with different autogenous vaccines [12–14], as they may use different adjuvants, bacterial concentrations as well as conditions used to make the pathogen grow and bacterial killing methods, among other variables. In addition, no field studies have evaluated the usefulness of an autogenous vaccine in the complete absence of antimicrobials on the farm.

Vaccination of gilts or sows (to elicit an enhanced passive maternal immunity) using autogenous vaccines is more commonly used in the field as this method is less costly than piglet vaccination. However, published studies on the use of autogenous vaccines in the field showed so far limited and/or no increase of passive maternal antibodies in piglets during the nursery barn period [11–13]. Two of these published field studies used vaccines manufactured within the same commercial vaccine company [12, 13]. Further research on length and duration of passive maternal immunity elicited by vaccines produced by different manufacturing companies is required. In the present study, the immune response, the clinical protection of piglets in the absence of any antimicrobial treatment as well as the effect on bacterial shedding of a three-dose multi-serotype *S. suis* autogenous vaccine (produced by a company different from that used in previous studies) and applied to gilts were evaluated.

## Materials and methods

### Farm selection and herd health status

A 1000 farrow-to-wean sow operation in Canada with external gilt replacement and no commingling was selected. Piglets were weaned at 3 weeks of age and transferred to a separate off-site, all-in-all-out, three-room nursery facility for additional 7 weeks. The farm experienced recurrent *S. suis* problems at the nursery site. Post-weaned mortality cumulated to 1.65%, with 30% being related to *S. suis*-associated diseases (laboratory confirmed, see below) in the presence of prophylactic, metaphylactic and curative antimicrobial treatments. This farm was selected with the objective to reduce not only mortality but also the use of antimicrobials. The operation had external gilt replacement from one single source and external gilts quarantined for 30 days upon arrival to the sow farm. Pre-trial health status was established as PRRSV positive (stable) and *Mycoplasma hyopneumoniae* negative. The sow farm herd was declared negative of PRRSV four months before the beginning of the trial. All external gilts were vaccinated against swine influenza, parvovirus, leptospirosis and erysipelas (Flusure XP; Farrowsure Gold^Ⓡ^) and porcine circovirus type 2 and *M. hyopneumoniae* (Circumvent PCV-MG2^Ⓡ^) at arrival into barn quarantine. Piglets received no vaccination at the farrowing barn.

Samples (meningeal swabs, joint swabs, heart, and brain) from nursery pigs were repeatedly submitted for complete diagnosis during at least 6 months prior to start the study. Serotyping of *S. suis* isolates was carried out at the diagnostic laboratory of the Faculty of Veterinary Medicine of the University of Montreal [17]. Final diagnosis of *S. suis* serotypes 2, 1/2, 5, 7 and 14-related diseases was established in this specific herd.

### Vaccine preparation and administration

Autogenous vaccine was prepared by a licenced company. It was composed of *S. suis* serotype 1/2 (strain 506), serotype 2 (strain 526), serotype 5 (strain 507), serotype 7 (strain 503), and serotype 14 (strain 541). The oil-based adjuvant used is owned by the company and no public information is available. In addition, detail on how the vaccine was prepared were not also available, as it usually happens with autogenous vaccines currently used in the field [12, 13]. The vaccine was administered concurrently (but not within the same injection) with another autogenous vaccine containing field strains of *Staphylococcus hyicus*, *Streptococcus dysglactiae* and *Staphylococcus aureus*. No ethical statement was required for the vaccine administration study as the protocol used was part of normal interventions in the farm and performed by the veterinarian in charge, as stated by the Animal Welfare Committee of the University of Montreal. For the blood collection for immunological studies and tonsil/saliva samples for quantitative PCR, the protocols and procedures were approved by the Animal Welfare Committee of the University of Montreal (protocol number Rech-2014).

### Immunization protocol

**Figure 1:**
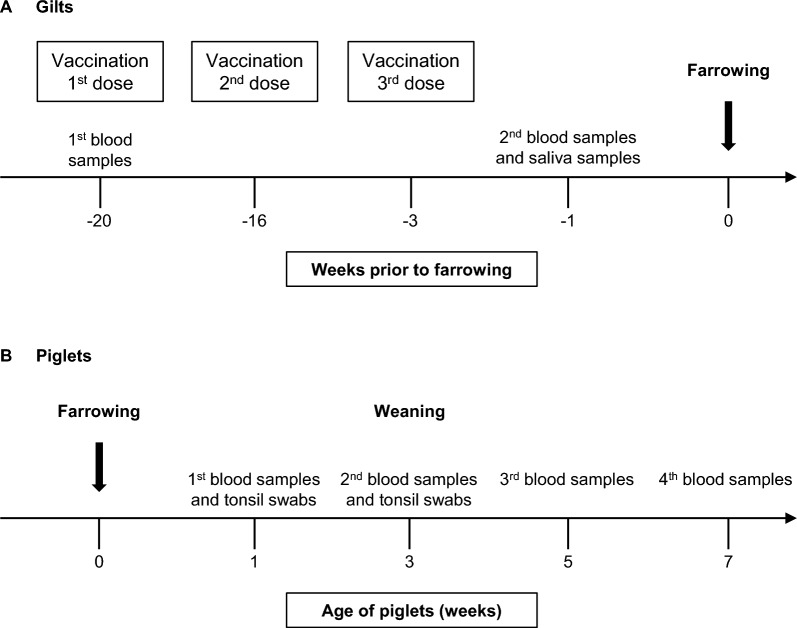
**Experimental design of field study.** **A** Gilts received three doses of an autogenous vaccine via intramuscular injection at −20 (pre-breeding), −16 and −3 weeks prior to farrowing. Blood samples were taken in all gilts prior to the first vaccination. Final blood samples and saliva samples were taken −1 week before farrowing. **B** Two piglets were randomly selected per litter from vaccinated and from non-vaccinated gilt groups for serological studies. Blood was taken from piglets at 1, 3, 5 and 7 weeks of age and tonsil swabs were taken from piglets at 1 and 3 weeks of age.

Out of the 70 gilts batch entered into the barn, vaccinated (*n* = 28) and non-vaccinated (*n* = 26) gilts were randomly selected. Gilts received three doses of the autogenous vaccine intramuscularly at 20 (pre-breeding), 16 and 3 weeks before farrowing (Figure 1A). It is important to mention that three doses of the autogenous vaccines for incoming gilts is a common practice in North America. Piglets from both vaccinated (*n* = 318) and non-vaccinated (*n* = 310) gilts were identified and enrolled in the trial (total *n* = 628). Of them, a total of 54 and 52 piglets from vaccinated and non-vaccinated gilts, respectively, were randomly tagged and numbered for serological follow up (Figure 1B). All piglets were weaned into four nursery rooms (two rooms with vaccinated piglets and two rooms with non-vaccinated piglets) in the same barn, with 20 piglets by pen. Other animals (not included in trial) were housed in the same facility.

### Blood and saliva/tonsil sampling

Blood samples were collected from gilts at −20 weeks (pre-breeding, before the first dose of the vaccine) and 1 week pre-farrowing (2 weeks after receiving 3rd dose of the vaccine) (Figure 1A). Piglets included in the serological study were sampled at 1 and 3 weeks in the farrowing room and at 5 and 7 weeks of age in the nursery barn (Figure 1B). After blood collection, serum was recovered and stored at −20 °C until analyses performed by enzyme-linked immunosorbent assay (ELISA) and by opsonophagocytosis assay (OPA) as described below.

Individual saliva samples (oral fluid collection via individual ropes) were collected at −1 week pre-farrowing for gilts (Figure 1A). Piglet tonsillar swabs were collected at 1 and 3 weeks of age (Figure 1B). Collected tonsillar and saliva samples were stored at −80 ℃ until analyzed by qPCR (for *S. suis* shedding) as described below.

### Enzyme-linked immunosorbent assay (ELISA)

Strains of *S. suis* used in the autogenous vaccine were also used as the coating antigen for ELISA Polysorb plates (Nunc-Immuno; Thermo Scientific, Mississauga, ON, Canada). The ELISA protocol was adapted from Corsaut et al. [13]. Briefly, bacteria were grown overnight onto 5% sheep blood agar plates at 37 ℃, and isolated colonies were cultured in 5 mL of Todd-Hewitt broth (THB) (Becton Dickinson, Mississauga, ON, Canada) for 8 h at 37 °C/5% CO_2_. Then, 10 µL of 1/1000 dilution of 8-h cultures were transferred into 30 mL of THB and incubated for 16 h at 37 °C/5% CO_2_. Stationary-phase bacteria were washed in phosphate-buffered saline (PBS) at pH 7.3. Bacterial pellet was then suspended in ddH_2_O and adjusted to a concentration equivalent to ~ 3 × 10^7^ CFU/mL. Plates were coated with 100 µL/well of the whole bacterial suspension, air-dried during two days at room-temperature (RT), and finally fixed with 50 µL/well of 100% methanol. After evaporation of methanol, plates were stored at RT until use. For titration of antibodies, plates were washed with PBS-tween (PBS-T), and treated with 300 µL of a blocking solution of 2% milk powder in PBS-T for 1 h at RT. Then 100 µL of different twofold based dilutions of pig sera (in PBS-T) were added to each well and incubated for 1 h at RT. For titration of porcine total Ig [IgG + IgM] or IgM, plates were incubated with peroxidase-conjugated goat anti-pig total Ig [IgG + IgM] (Jackson ImmunoResearch, West Grove, PA) or IgM (BioRad, Mississauga, ON, Canada) antibodies, respectively, for 1 h at RT. For porcine IgG1 or IgG2 detection, mouse anti-porcine IgG1 or IgG2 (BioRad) was added for 1 h at RT. After washing, peroxidase-conjugated goat anti-mouse IgG (Jackson ImmunoResearch) was added for 1 h at RT. Plates were developed with 3,3’,5,5’-tetramethylbenzidine (TMB; Invitrogen, Burlington, ON, Canada) substrate and the enzyme reaction was stopped by addition of 0.5 M H_2_SO_4_. Absorbance was read at 450 nm with an ELISA plate reader (Biotek, Santa Clara, CA, USA). The reciprocal of the last serum dilution that resulted in an optical density at 450 nm (OD_450_) of ≤ 0.2 (cut-off) was considered the titer of that serum. To control inter-plate variations, an internal reference positive control was added to each plate. This positive control was composed by a pool of serum of ten sows randomly selected on farm that showed high ELISA values against all 5 vaccine strains (serotypes 1/2, 2, 5, 7 and 14) because of their natural exposition to these serotypes on farm. Reaction in TMB was stopped when an OD_450_ of 1.0 was obtained for the positive internal control. Optimal dilutions of the positive internal control sera and anti-porcine antibodies or conjugates were determined during preliminary standardization assays. It should be noted that ELISA tests evaluated only whether or not levels of antibodies significantly increased; however, it is difficult to ascertain in some cases if such increase has a biological relevance.

### Opsonophagocytosis assay (OPA)

The OPA test was performed as previously published [13], with some modifications. The *S. suis* serotype 2 strain was used for this protocol as a representative serotype for the study and the capacity of passive antibodies to kill bacteria was evaluated in piglets at 1, 3 and 5 weeks of age coming from either vaccinated or non-vaccinated gilts. Whole blood of 4–8-week-old piglets (in the absence of significant levels of maternal antibodies) coming from a high health status herd was used as a source of phagocytic cells. These piglets originated from a farm without *S. suis* endemic infection and blood was intravenously collected in vacutainer sodium heparin tubes (Becton, Dickinson, Franklin Lakes, NJ, USA), and kept at room temperature. Using washed bacterial cultures grown as described above, final bacterial suspensions were prepared in complete cell culture medium (RPMI 1640 supplemented with 5% heat-inactivated fetal bovine serum, 10 mM HEPES, 2 mM L-glutamine and 50 µM 2-mercaptoethanol; Invitrogen) to obtain a concentration of 1 × 10^6^ CFU/mL. The number of CFU/mL in the final suspension was determined by plating samples onto Todd-Hewitt agar (THA). Whole blood (containing approximately 1 × 10^10^ leukocytes/mL) was mixed with the *S. suis* suspension to obtain a multiplicity of infection (MOI) of 0.01. Control and sample sera from immunized animals were added to a concentration of 20% *v*/*v* in microtubes to a final volume of 200 µL. Control sera came from naïve pigs (absorbed against different serotypes of *S. suis* and presenting negative ELISA values), and positive sera were obtained and pooled from sows (originated from the same farm and presenting high ELISA values). The tube tops were pierced using a sterile needle and incubated for 2 h at 37 °C with 5% CO_2_, with gentle agitation. After incubation, viable bacterial counts were performed on THA using a spiral plater (Whitley Automated Spiral Plater, Whitley Wasp Touch, Frederick, MD). The percentage of bacterial killing was determined using the following formula:

$${\text{\% Bacteria killed = [1}}\, - \,{\text{(bacteria recovered from sample tubes/bacteria recovered from negative control tube with control serum)] }} \times { 100}$$

### Quantification of total *S. suis* and *S. suis* serotypes 2 (and 1/2), 5, 7 and 14 (and 1) shedding

The qPCR was used to measure total *S. suis* and *S. suis* serotypes 2 (and 1/2), 5, 7 and 14 (and 1) shedding in gilts as well as in 1- and 3-week-old piglets. Serotypes 2 and 1/2 as well as serotypes 1 and 14 cannot be differentiated by PCR. Tonsil and saliva samples were centrifuged at 21 000 × *g* and the supernatant was removed. Pellets were then treated with lysozyme in 200 µM Tris HCl-EDTA-triton for 30 min at 37 °C. QIAamp DNA kit (Qiagen, Toronto, ON, Canada) was used to extract DNA following the manufacturer instructions. The qPCR was used to quantify the concentration of total *S. suis* as well as that of *S. suis* serotypes [2 (and 1/2), 5, 7 and 14 (and 1)] from tonsil swab/saliva samples. The qPCR was performed using the EXOone *Streptococcus suis* oneMIX qPCR kits from Exopol (San Mateo de Gallego, Zaragoza, Spain) following the manufacturer’s instructions.

### Clinical evaluation of piglets

Clinical signs, mortality and euthanasia from all enrolled piglets were recorded by farm staff daily. Pigs were identified at birth by ear tag colour and/or number and were followed until the end of the nursery period (10 weeks of age). Clinical signs related to “*S. suis*-associated diseases” [7] were listed as: arthritis, meningitis, and sudden death. It should be noted that these clinical signs are not exclusively induced by *S. suis*, since other pathogens, such as *Glaesserella parasuis*, *Escherichia coli*, *Actinobacillus suis* and *Erysipelothrix rhusiopathiae* may induce some or most of the these clinical signs; indeed, necropsy and bacteriological analysis must be done to identify the pathogen(s) involved [1]. Antibiotic treatments, including preventive, metaphylactic (still allowed in North America) and curative were removed during the trial. Previous to the study, penicillin was routinely used in the nursery. Animals showing clinical signs were immediately euthanized by farm staff. After euthanasia, meningeal swabs, joint swabs and spleen tissue samples were collected from all piglets presenting clinical signs related to *S. suis*-associated diseases [7] and submitted for culture to identify the pathogen involved. Culture was carried out at the diagnostic service of the University of Montreal. Isolation was performed by culture on blood agar, identification of the bacteria was done by MALDI-TOF [18] and confirmation by *rec-N* PCR [19]. In cases where *S. suis* isolates were identified, these were further serotyped using a multiplex-PCR [20]. Serotype 2 and 1/2 isolates were differentiated using a mismatch amplification mutation PCR assay [17].

### Statistical analyses

ELISA data were log-10 transformed to normalize distributions. Unless otherwise specified, a linear mixed model was used with sampling time as the within-subject fixed effect, group (vaccinated or non-vaccinated) as the between-subject fixed effect, and animal identification (id) as random effect. A priori contrasts were performed to compare pairs of means adjusting the alpha level downward for each comparison with the sequential Benjamini–Hochberg procedure. In the analysis of IgG1 and IgG2 subclasses, equal variance *t*-test was used to compare means according to the vaccinal status. For OPA analysis, data were arcsine square-root transformed to normalize distributions. Statistical analyses were performed using SAS 9.4 (SAS, Cary, NC, USA). The level of statistical significance was set at 0.05.

## Results

### Vaccination in gilts increased total Ig, IgG1 and IgG2, but not IgM, antibody levels

**Figure 2:**
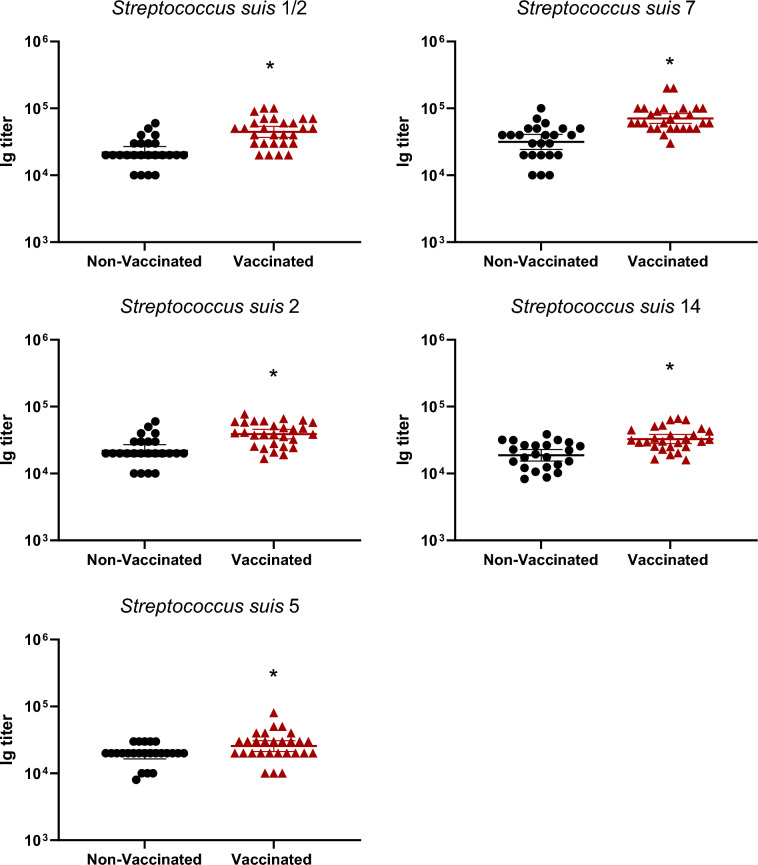
**Immunoglobulin (Ig) levels in gilts against *S. suis* serotypes 1/2, 2, 5, 7, and 14.** Ig [IgG + IgM] titers were determined by ELISA on serum samples collected post-vaccination (Figure 1A), against all five vaccine strains individually. Antibody titers for individual gilts are shown with horizontal bars representing geometrical mean ± standard error of mean (SEM). Significant values (*p* < 0.05) are shown with asterisks.

**Figure 3:**
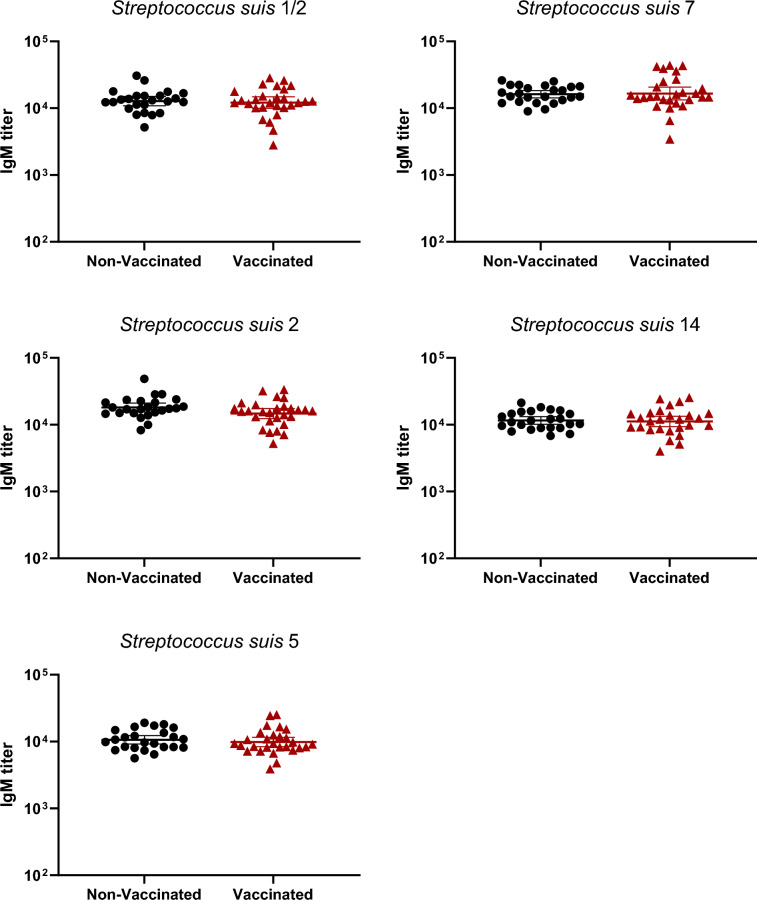
**IgM isotype antibodies raised in gilts after vaccination against *S. suis* serotypes 1/2, 2, 5, 7, and 14.** IgM titers were determined by ELISA on serum samples collected post-vaccination (Figure 1A), against all five vaccine strains individually. Antibody titers for individual gilts are shown with horizontal bars representing geometrical mean ± standard error of mean (SEM).

**Figure 4:**
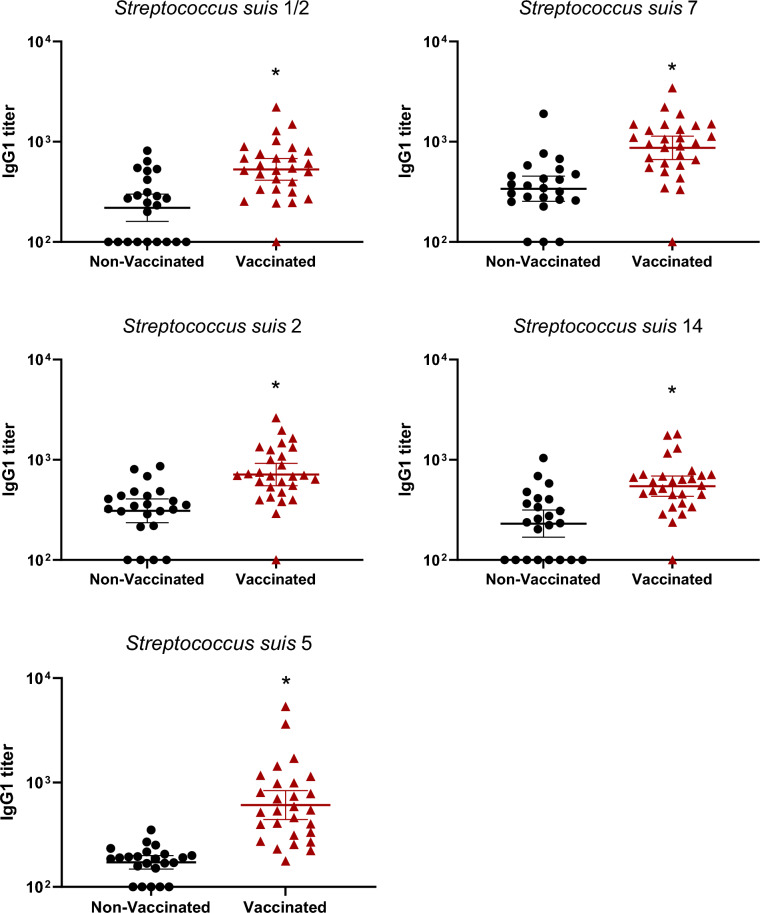
**IgG1 subclass antibodies raised in gilts after vaccination against *S. suis* serotypes 1/2, 2, 5, 7, and 14.** IgG1 titers were determined by ELISA on serum samples collected post-vaccination (Figure 1A), against all five vaccine strains individually. Antibody titers for individual gilts are shown with horizontal bars representing geometrical mean ± standard error of mean (SEM). Significant values (*p* < 0.05) are shown with asterisks.

**Figure 5:**
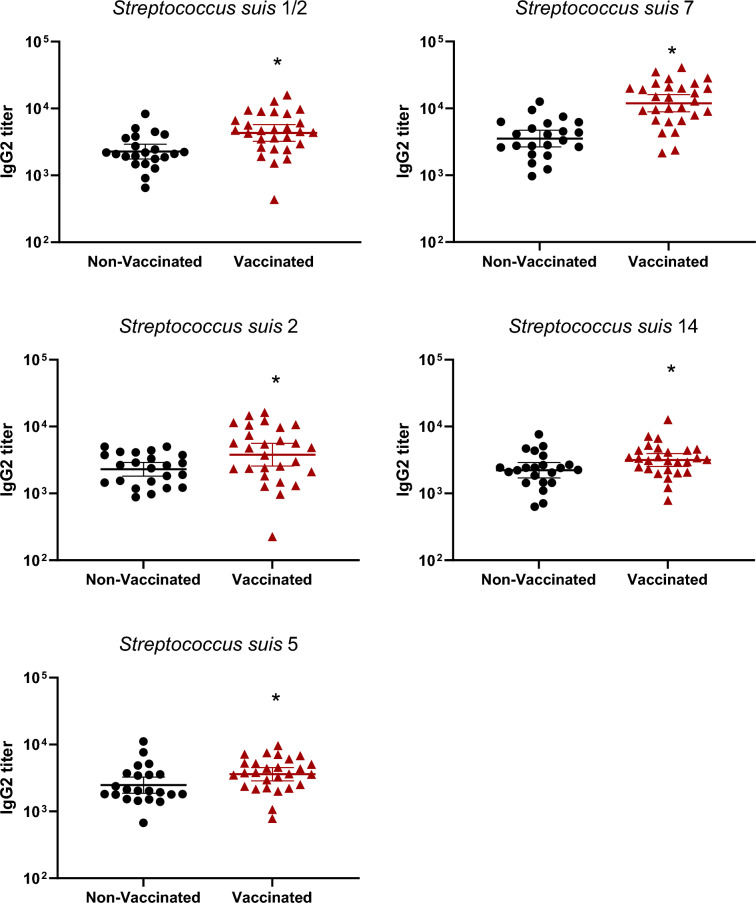
**IgG2 subclass antibodies raised in gilts after vaccination against *S. suis* serotypes 1/2, 2, 5, 7, and 14**. IgG2 titers were determined by ELISA on serum samples collected post-vaccination (Figure 1A), against all five vaccine strains individually. Antibody titers for individual gilts are shown with horizontal bars representing geometrical mean ± standard error of mean (SEM). Significant values (*p* < 0.05) are shown with asterisks.

The autogenous vaccine contained five serotypes of *S. suis*: 2, 1/2, 5, 7 and 14. In non-vaccinated gilts, levels of immunoglobulins (Ig) [IgG + IgM] in gilts against those serotypes were already high (around à titer of 10^4^ or higher; Figure 2). After three doses of the vaccine, titers became significantly higher in vaccinated groups compared to the non-vaccinated controls (Figure 2). In addition to the increase of Ig against *S. suis* tested serotypes, the goal of an efficient vaccine is also to obtain isotype switching from IgM to IgG subclasses. In this regard, the level of IgM antibodies did not change after vaccination, with very high levels already present in control animals against all serotypes tested (Figure 3). In contrast, a clear increase of IgG1 and IgG2 levels after three vaccine-doses was observed and this, for all serotypes tested, when compared to control animals (*p* < 0.05) (Figures 4 and 5).

### Maternal antibody transfer to piglets increased after gilt vaccination and lasted until 5 weeks of age for most serotypes

**Figure 6:**
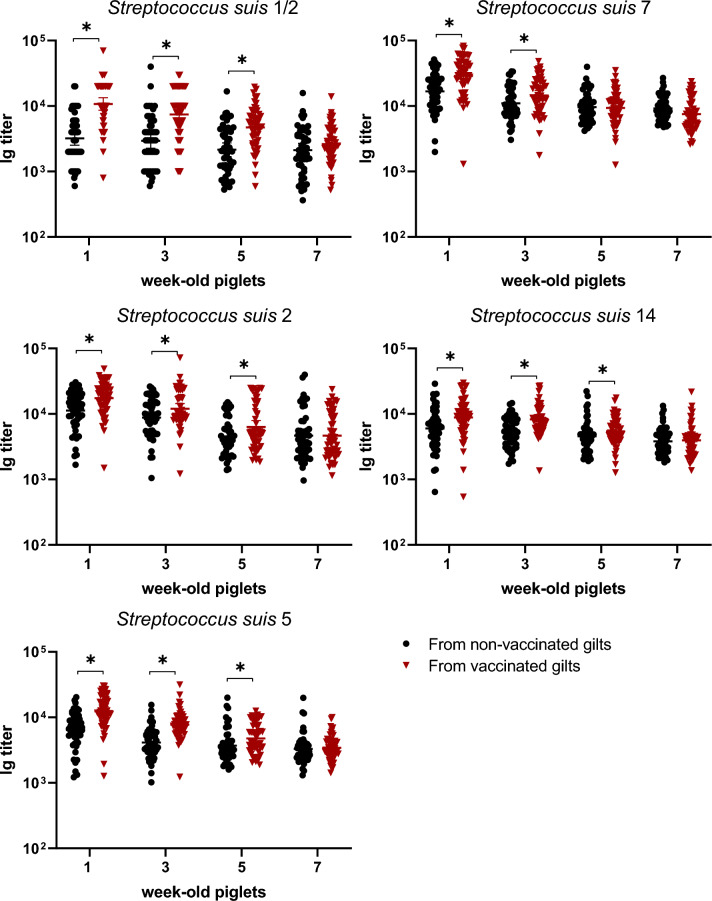
**Kinetics of Ig in piglets against* S. suis* serotypes 1/2, 2, 5, 7, and 14 born from either vaccinated or non-vaccinated gilts.** Two piglets per litter were randomly selected and assigned to vaccinated or non-vaccinated groups depending on the vaccination status of their gilts. Piglets were sampled at 1, 3, 5, and 7 weeks of age (Figure 1B). Ig [IgG + IgM] titers were determined by ELISA against all five vaccine strains individually. Antibody titers for individual piglets are shown with horizontal bars representing geometrical mean ± standard error of mean (SEM). Significant values (*p* < 0.05) are shown with asterisks.

**Figure 7:**
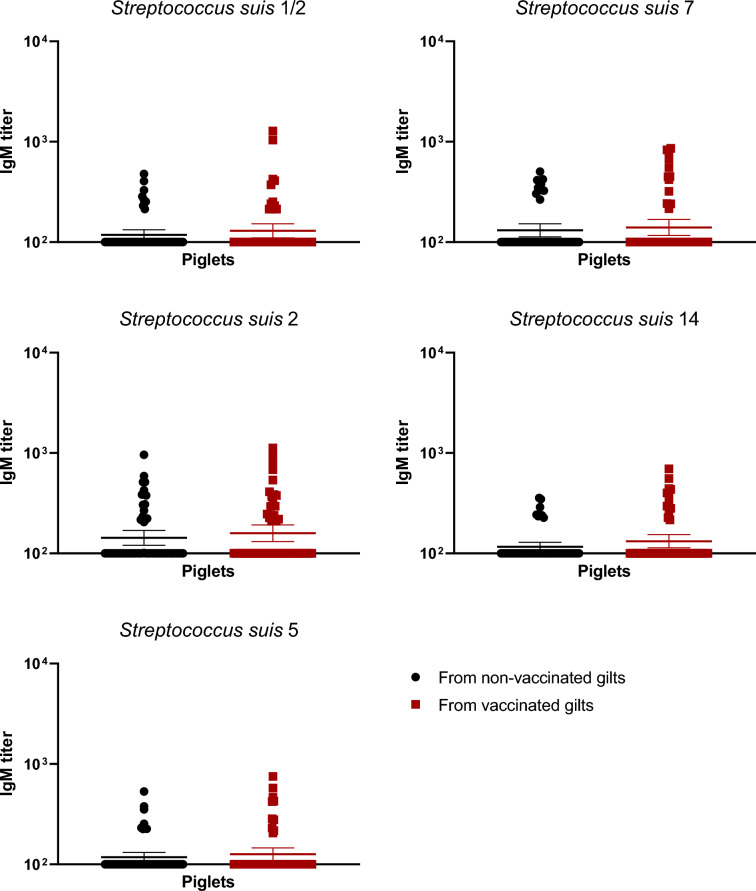
**IgM isotype antibodies in piglets at 1 week of age against* S. suis* serotypes 1/2, 2, 5, 7, and 14 born from either vaccinated or non-vaccinated gilts.** Two piglets per litter were randomly selected and assigned to vaccinated or non-vaccinated groups depending on the vaccination status of their gilts. IgM titers were determined by ELISA against all five vaccine strains individually in serum samples at 1 week of age. Antibody titers for individual piglets are shown with horizontal bars representing geometrical mean ± standard error of mean (SEM).

**Figure 8:**
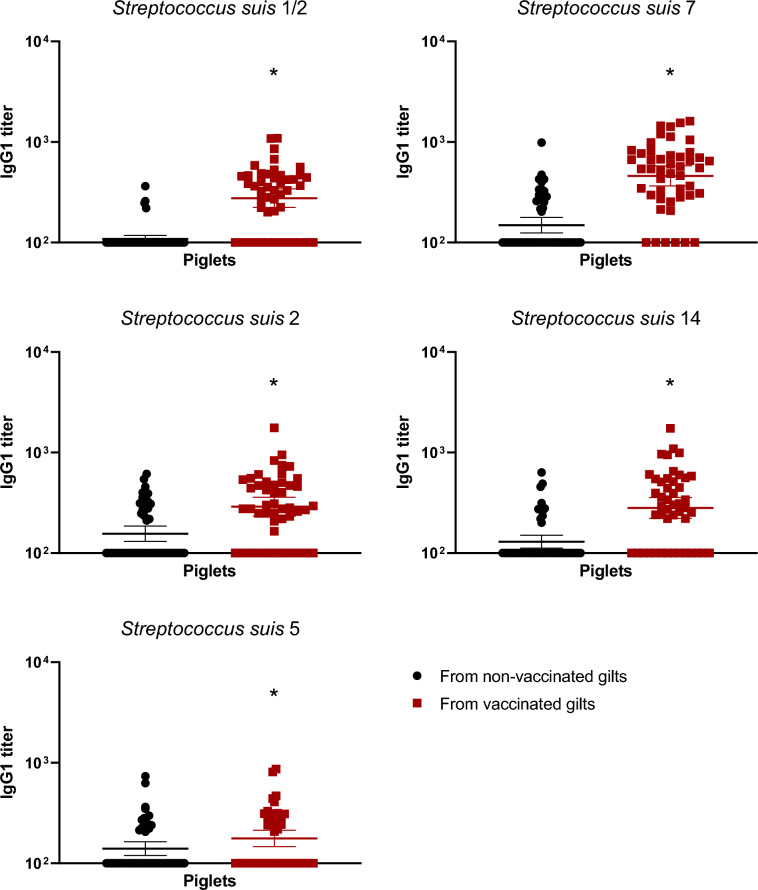
**IgG1 subclass antibodies in piglets at 1 week of age against* S. suis* serotypes 1/2, 2, 5, 7, and 14 born from either vaccinated or non-vaccinated gilts.** Two piglets per litter were randomly selected and assigned to vaccinated or non-vaccinated groups depending on the vaccination status of their gilts. IgG1 titers were determined by ELISA against all five vaccine strains individually in serum samples at 1 week of age. Antibody titers for individual piglets are shown with horizontal bars representing geometrical mean ± standard error of mean (SEM). Significant values (*p* < 0.05) are shown with asterisks.

**Figure 9:**
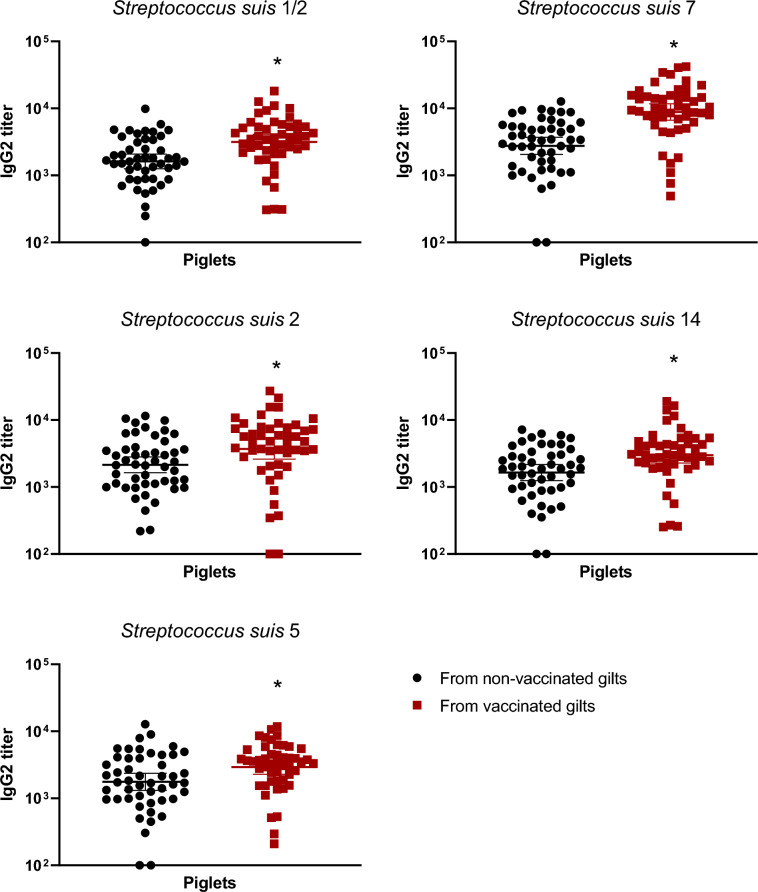
**IgG2 subclass antibodies in piglets at 1 week of age against* S. suis* serotypes 1/2, 2, 5, 7, and 14 born from either vaccinated or non-vaccinated gilts.** Two piglets per litter were randomly selected and assigned to vaccinated or non-vaccinated groups depending on the vaccination status of their gilts. IgG2 titers were determined by ELISA against all five vaccine strains individually in serum samples at 1 week of age. Antibody titers for individual piglets are shown with horizontal bars representing geometrical mean ± standard error of mean (SEM). Significant values (*p* < 0.05) are shown with asterisks.

The goal of a sow vaccination program is to increase maternal antibodies of their offspring via colostrum intake. As higher levels of anti-*S. suis* Ig [IgG + IgM] were observed in gilts for all serotypes, significantly higher levels of maternal antibodies could be detected in piglets from vaccinated gilts until 5 weeks of age for all serotypes, with the exception of serotype 7 (Figure 6). In the latter case, statistically significant differences with piglets from non-vaccinated gilts were observed until 3 weeks of age only (Figure 6). Isotype profile was evaluated at one week of age, corresponding to the peak of maternal antibodies transferred to the litters. IgM titers for all serotypes were low and similar between the vaccinated and non-vaccinated groups (Figure 7). The predominant isotype profiles were both IgG1 and IgG2 in piglets born from vaccinated gilts and this, for all serotypes (Figures 8 and 9).

### Vaccination of gilts with the autogenous bacterin partially increased the killing capacity of antibodies of their offspring until the third week of age

**Figure 10:**
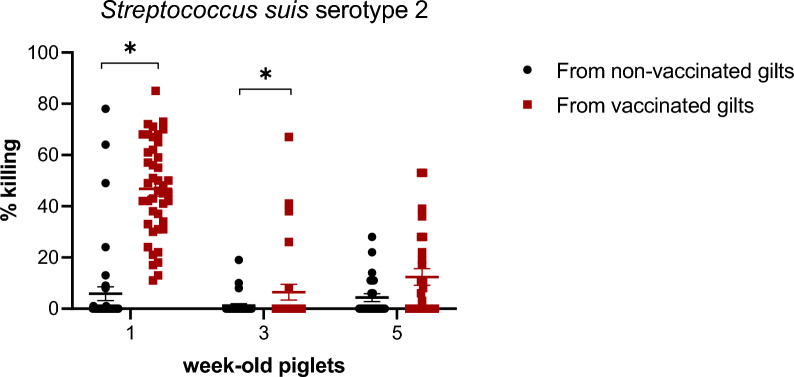
**Opsonophagocytosis of *S. suis* serotype 2 induced by serum antibodies from piglets.** Two piglets per litter were randomly selected and assigned to vaccinated or non-vaccinated groups depending on the vaccination status of their gilts. Blood samples were collected at 1, 3 and 5 weeks of age (Figure 1B) to evaluate antibody functionality in the opsonophagocytosis assay. Results are expressed at % of bacterial killing of individual sera, with horizontal bars representing mean ± standard error of mean (SEM). Significant values (*p* < 0.05) are shown with asterisks.

Functionality of the antibodies in both groups was evaluated using serotype 2 as a model. As shown in Figure 10, OPA activity of maternal antibodies in piglets at 1 week of age was clearly different and higher in animals from vaccinated gilts when compared to those coming from non-vaccinated gilts. Still, killing capacity of individual piglets varied from 10 to 85%. An increased capacity of bacterial killing in piglets coming from vaccinated gilts was still significant at 3 weeks of age, but not at 5 weeks of age, although a tendency of a higher OPA activity was still observed at the latter age (Figure 10).

### Vaccination had no effect on *S. suis* shedding

**Figure 11:**
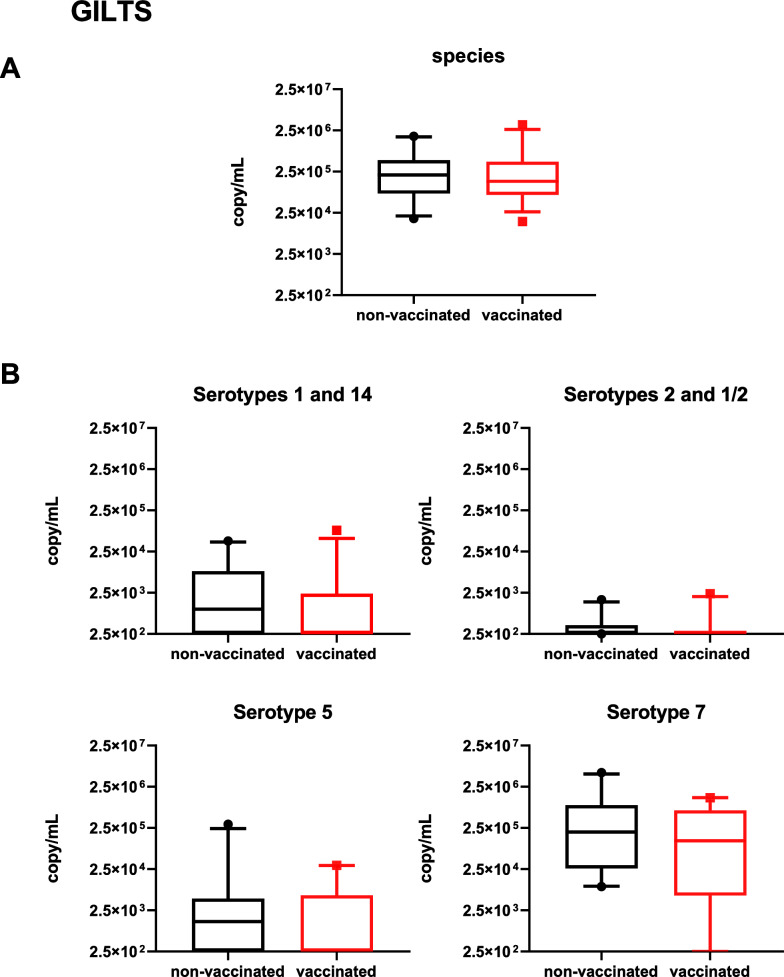
**Total *S. suis* species (A) and serotype specific qPCR (B) in gilts.** Saliva samples were collected at −1 week before farrowing from vaccinated and non-vaccinated gilts (Figure 1A) to evaluate if the vaccination program influenced potential bacterial shedding. Results are expressed in copies/mL per sample. The minimum value of Y axe is set at 2.5 × 10^2^ copy/mL, corresponding to the detection limit of the qPCR test. Data are presented in a box-and-whisker diagram with the lower whisker being drawn at the 5th percentile and the upper whisker being drawn at the 95th percentile.

**Figure 12:**
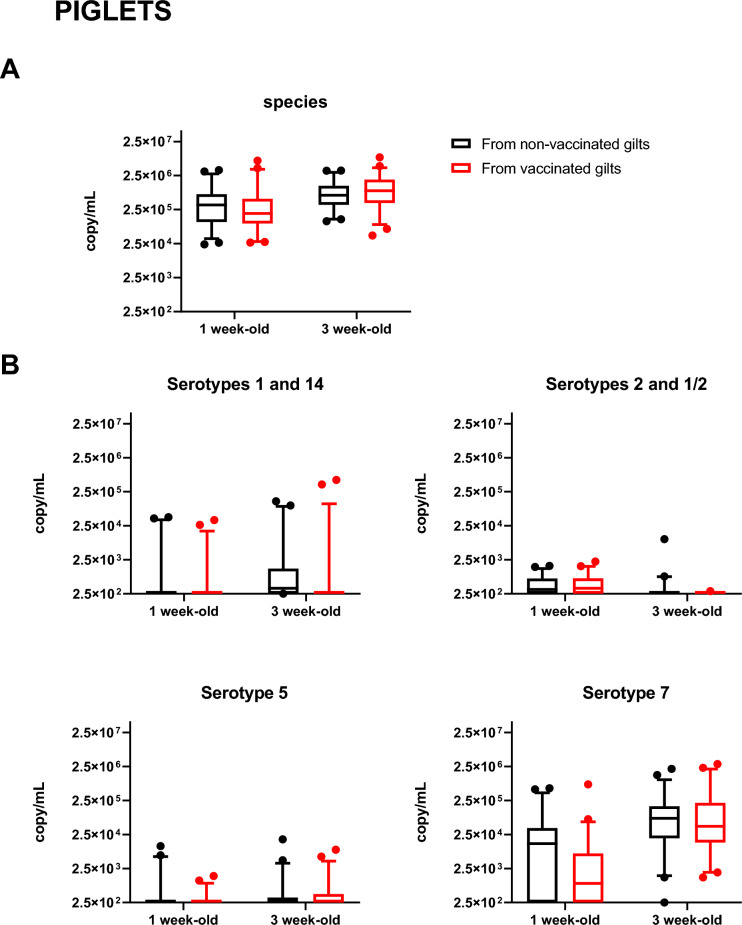
**Total *S. suis* species (A) and serotype specific qPCR (B) in piglets.** Two piglets per litter were randomly selected and assigned to vaccinated or non-vaccinated groups depending on the vaccination status of their gilts. Tonsil samples were collected at 1 and 3 weeks of age (Figure 1B). Results are expressed in copies/mL per sample. The minimum value of Y axe is set at 2.5 × 10^2^ copy/mL, corresponding to the detection limit of the qPCR test. Data are presented in a box-and-whisker diagram with the lower whisker being drawn at the 5th percentile and the upper whisker being drawn at the 95th percentile.

Another goal of the study was to evaluate if the vaccination program was able to reduce *S. suis* (and/or specific serotypes) potential shedding either by gilts, piglets or both. Using qPCR, Figures 11, 12A show results on total *S. suis* species in gilt saliva or piglet tonsils, respectively. As expected, all animals (gilts and piglets) were highly colonized by *S. suis*. Furthermore, piglet levels of total *S. suis* species slightly increased with age (Figure 12A). In gilts, levels of individual serotypes vary, with high levels of serotype 7, intermediate levels of serotypes 1 (and/or 14) and 5, and very low levels of serotype 2 (and/or 1/2) (Figure 11B). In piglets, the number of individual serotypes detected in tonsils was lower than that observed in gilt saliva. However, serotype 7 predominated, as observed in gilts (Figure 12B).

We next analyzed the effect of vaccination. Vaccinated gilts did not show a significant decrease of *S. suis* bacterial load (copies/mL) when compared to the non-vaccinated gilts (Figure 11A). Similar results were observed in piglets at both, 1 and 3 weeks of age (Figure 12A). When evaluating specific serotypes, although vaccination seemed to reduce bacterial shedding in gilts for serotypes 5, 7 and 1 (and/or 14) (Figure 11B), differences were not significant. No differences were also observed for the individual serotypes in piglets (Figure 12B). In conclusion, gilt vaccination did not significantly influence *S. suis* shedding.

### No *S. suis* outbreak identified on farm, yet *S. suis*-associated clinical signs were found

There was no mortality from birth to weaning in the selected group of piglets in this study. During the trial, nursery animals presenting clinical signs related to *S. suis*-associated diseases were immediately euthanized, necropsied and submitted for bacterial examination. The overall mortality considered to be related to *S. suis*-associated disease (based on clinical signs) was 2.22%, with 0.31% in the vaccinated group and 4.19% in the non-vaccinated group (Table 1). Indeed, although no antibiotics were present in the farm, only 14 animals died: ten of sudden death/septicemia and four with either meningitis and/or arthritis. However, mortality was mainly not due to *S. suis* as revealed by results from the diagnostic laboratory. In the vaccinated group, one non-typable isolate of *S. suis* was recovered (0.31%). In the non-vaccinated group, *S. suis* was isolated from 3 animals (two serotype 2 and one non-typable), which represents 0.96%. The non-typable isolates were recovered with other contaminants (probably post-mortem invasion), so their role as the causative agent could not be confirmed. The additional 10 piglets died from other diseases that were not related to *S. suis*. Indeed, *Erysipelothrix rhusiopathiae* was identified in six of these piglets. Thus, no *S. suis* outbreak could be identified during the time of the trial on this farm, limiting the ability to properly measure vaccine efficacy.

**Table 1:** **Distribution of confirmed *S. suis* mortality during the nursery period among 318 vaccinated and 310 non-vaccinated piglets included in the trial**^**1**^

Piglets	Dead/euthanized pigs with *S. suis*-associated diseases (No of pigs/percentage of total pigs in each group)	Dead/euthanized pigs with confirmed isolation of* S. suis* (No of pigs/percentage of total pigs in each group)
From vaccinated gilts	1 (0.31%)	1 (0.31%)
From non-vaccinated gilts	13 (4.20%)	3 (0.96%)
Total number of piglets	14 (2.30%)	4 (0.60%)

^1^A total of 318 and 310 piglets from vaccinated and non-vaccinated gilts, respectively, were followed clinically. *S. suis*-associated diseases was defined as sudden death, arthritis and/or meningitis.

## Discussion

Autogenous vaccines are very popular amongst swine producers, as they may represent an interesting alternative to antimicrobials. There is no current efficacious and multivalent commercial vaccine available for *S. suis* control. Most published studies on bacterins used laboratory-made vaccines with a formulation that may be far from those used in the field [6, 11, 16, 21–23]. To the best of our knowledge, only four field studies (one of them performed 25 years ago) are available on the immunogenicity and/or clinical protection efficacy of autogenous bacterins manufactured by licensed companies [12–15]. At least two of the recent trials used vaccines produced by the same company [12, 13]. In the first one, a two-dose vaccine program containing *S. suis* serotypes 7 and 9 was applied either to gilts or piglets. Results showed that, despite a higher anti-*S. suis* levels in vaccinated gilts before farrowing, their piglets did not show higher maternal antibody levels against both serotypes when compared to those from non-vaccinated gilts [13]. Vaccination of young piglets (coming from non-vaccinated gilts) did not induce any seroconversion [13]. In the second study, using a three-dose autogenous vaccine program in gilts containing another *S. suis* serotype 7 strain, higher antibody levels were observed not only in vaccinated gilts but also in 7-day old piglets [12]. However, at 18 days of age, levels of antibodies in piglets significantly dropped and were similar to those from piglets derived from non-vaccinated gilts, leaving animals unprotected at the riskiest period in the nursery [12].

Manufacturing companies may use different laboratory protocols (growth conditions, media, etc.), adjuvants (types and final concentration), bacterial inactivation techniques as well as different bacterial concentrations [11]. Indeed, such procedures and compositions are, in general, part of confidential information and there are no standardized protocols that must be officially followed by these companies. As some of the previously mentioned studies contained autogenous vaccines that were manufactured by the same licensed manufacturing company, it is unknown if similar results are obtained when the product is produced by a different company. In the current study, a multi-serotype autogenous vaccine manufactured from a different vaccine company than that previously evaluated [12, 13] was used. The immunological characterization of the antibody response confirmed that the basal antibody level before vaccination against *S. suis* in adult animals is high—including elevated titers of IgM, as previously shown [12, 13, 21, 24]. This can be explained by natural exposure of these animals to *S. suis* (and probably *S. suis*-like microorganisms sharing common epitopes with *S. suis*) present in the farm. Indeed, saliva samples taken from gilts revealed high bacterial loads of *S. suis* in most animals. After three doses of the autogenous vaccine, Ig [IgG + IgM] antibody levels against *S. suis* in vaccinated gilts significantly increased for all five serotypes when compared to control group. Isotyping switching was clearly observed for all serotypes included in the vaccine with concomitant no changes in IgM levels. Isotype switching from IgM to IgG1 and IgG2 [13] or from IgM to IgG1 only [12] might vary upon vaccine formulation and has the potential to affect clinical protection in the piglets [25, 26].

Different from what was previously published with an autogenous vaccine produced by another company [12, 13], the increased response seen in gilts was sufficient to improve maternal antibody transfer to piglets up to 5 weeks of age for all serotypes, with the exception of serotype 7, for which a higher level of maternal antibodies last for 3 weeks only. As observed in gilts and similarly to what was described by Corsaut et al. [13], both IgG1 and IgG2 subclasses were present in piglets, with low levels of IgM. As mentioned above, it is very difficult to point out the exact reasons that may explain why maternal antibodies last longer using this autogenous vaccine when compared the previous ones [12, 13]. Both, the conditions used for the production of this autogenous vaccine and the use of a different adjuvant may have probably played an important role. It has been reported that by changing the adjuvant only, a bacterin prepared under exactly the same conditions may be either highly immunogenic and protective or low immunogenic and not protective at all [16]. As mentioned, a multi-serotype autogenous vaccine was used in the current study, a very common practice in North America (M. Gottschalk, unpublished observations), where usually more than one serotype is recovered in affected farms [13]. In addition to the adjuvant, another hypothesis that can be raised is that adding several serotypes may increase the level of antibodies generated to common antigens. However, we have observed that a monovalent experimental bacterin induces similar levels of antibodies against a given serotype than a multivalent (5 serotypes) vaccine (M. Gottschalk and M. Segura, unpublished data). Finally, a relatively high variation in the level of maternal antibodies among the individuals could be observed at 1 week of age. This has been previously observed in other studies [13, 24] and could be explained by differences in colostrum uptake/adsorption among piglets.

It should be noted that, although a statistically significant difference in antibody levels was observed in most piglets from vaccinated gilts at 5 weeks of age when compared to those from non-vaccinated gilts, it is unknown if such difference has a biological relevance and induces protection. Although the clinical protection could not be evaluated (see below), we used the OPA test, previously used as a surrogate marker for protection [13, 27], to evaluate a potential capacity of protection of antibodies generated by the autogenous vaccine. Results obtained with this test and the serotype 2 (as a representative serotype) showed that a higher opsonophagocytosis capacity of maternal antibodies in piglets coming from vaccinated gilts could be observed at 1 and 3 weeks of age. This is the first time that a difference between piglets from vaccinated gilts or non-vaccinated gilts is observed with the OPA test [13]. At 5 weeks of age, this difference was no longer significant, indicating probably that the protection from maternal antibodies would not last during all the nursery period. Indeed, in many farms, clinical problems and death associated to *S. suis* occurs between 5 and 10 weeks of age (late nursery) [1]. In such cases, a three-dose vaccination program with an autogenous vaccine such as the one evaluated herein would not induce a sufficient high level of antibodies to cover such a period. Indeed, vaccination of gilts/sows may be useful when young piglets in the farrowing unit or early in the nursery are affected.

Although the current and previous studies with control groups could not demonstrate that maternal antibodies last for the whole nursery period [12, 13, 21], practitioners still recommend sow vaccination with autogenous vaccines [28]. Other than protective passive antibodies, we hypothesized that the autogenous vaccine may have a potential effect of reducing *S. suis* shedding (either total *S. suis* or the specific serotypes included in the vaccine). To validate this hypothesis, saliva samples of gilts before farrowing were taken. It is accepted that *S. suis* is normally present not only in tonsils [1], but also in saliva, being the latter one of the principal reservoir for this bacterial pathogen [29]. Results showed that although vaccination increase antibody titers, it did not reduce *S. suis* presence in saliva of gilts, neither total *S. suis* or that of serotypes tested. Similar results were observed with tonsil samples from piglets at 1 and 3 weeks of age, where level of *S. suis* and the serotypes included in the vaccine were similar between animals derived from vaccinated or non-vaccinated gilts. As expected, the total number of *S. suis* was, in general, higher than those of specific serotypes, an observation that was previously reported [29].

The evaluation of the impact of the application of an autogenous vaccine program on the development of *S. suis*-associated diseases in the field is not an easy task. Indeed, other pathogens may induce similar pathologies [7], the use of antimicrobials may prevent the development (and etiological diagnosis) of clinical signs and, finally, bacteriological follow up of clinical cases is rarely done in a systematic way in most studies. To our knowledge, there are no previous studies that eliminated the use of antimicrobials during the evaluation of an autogenous vaccine program on a farm, as it was done in the current study. In addition, *S. suis*-associated disease cases were sent for confirmatory necropsy, followed by bacteriology and *S. suis* serotyping (if present) for all clinical cases. Unfortunately, the clinical protective effect of the vaccination program with the autogenous vaccine could not be evaluated. Indeed, although some *S. suis*-associated clinical signs could be observed, only limited confirmed-*S. suis* cases were identified during trial. The spontaneous disappearance of *S. suis* cases at the moment of the autogenous vaccine application has previously been reported, and the cause(s) remain(s) unknown [24]. In the current study, the fact that the herd became negative for PRRSV just before the beginning of the trial may have positively influenced the lack of clinical diseased caused by *S. suis*, although this is only a hypothesis. *E. rhusiopathiae* was mainly identified as the most common pathogen recovered mostly from cases of sudden death and arthritis, despite the fact that the farm used sow vaccination against that pathogen, and there were no reports on its isolation from diseased piglets in the last years. It is possible that elimination of all antimicrobial treatments in the farm during the trial predisposed the appearance of such a pathogen. Results of the current study reinforce the need of having always a non-vaccinated control group, removing antimicrobial treatments on the farm and etiological confirmation of clinical cases done by a diagnostic laboratory. Indeed, relying on clinical observations (even in the presence of a control group) or evaluation of an autogenous vaccine comparing pre- and post-application in a farm (a very common practice in the field) could be highly misleading, due to the cycling appearance of *S. suis* disease and the difficulties on the assessment of a differential diagnosis with other aetiological agents.

Results of the current study showed that the autogenous vaccine tested in the current study induced higher levels of antibodies than those previously reported [12, 13]. Although the multi-serotype autogenous vaccine tested in a three-dose program in gilts induced passive antibodies which last up to 5 weeks of age in piglets (longer than previously available data), functional activity of these antibodies only last until weaning age (3 weeks), leaving older nursery piglets susceptible to disease. Though vaccination of gilts/sows is very popular in the field, there is still no study clearly showing that this approach would protect the whole period at risk for *S. suis* disease. In addition, it was shown that these antibodies did not have any influence on *S. suis* shedding. While clinical protection could not be evaluated due to the lack of real *S. suis* cases during the trial, this study strengths the need of a complete evaluation of the clinical protection that must include laboratory confirmation of the aetiological agent involved in the presence of *S. suis*-associated clinical signs. Indeed, there is a need for future field trials, to always include a non-vaccinated control group, to eliminate if possible any antimicrobial treatment in the farm and to use diagnostic laboratory when evaluating the protective effect of autogenous vaccines.

## Data Availability

The data presented in this study are available on request from the corresponding author.

## Abbreviations

CFU: colony-forming unit
ELISA: enzyme-linked immunosorbent assay
Ig: immunoglobulin
IgG: immunoglobulin G
IgM: immunoglobulin M
PCR: polymerase chain reaction
qPCR: quantitative PCR
THA: THB agar
THB: Todd Hewitt broth
PRRSV: porcine reproductive and respiratory syndrome virus
OPA: opsonophagocytosis assay
TMB: 3,3’,5,5’-Tetramethylbenzidine
